# Supplementary material for: Strongyloides stercoralis infection induces gut dysbiosis in chronic kidney disease patients
Source: PLoS Negl Trop Dis. 2022 Sep 6;16(9):e0010302. doi: 10.1371/journal.pntd.0010302 (PMC9481163; doi:10.1371/journal.pntd.0010302)
Supplement: Attachment — Submitted filename: Response to Reviewers PNTD-D-22-00290R1.docx [file pntd.0010302.s001.docx]

**Response to reviewers**

PNTD-D-22-00290."*Strongyloides stercoralis* infection induces gut dysbiosis in chronic kidney disease patients"

We thank the reviewer and editor for their critical evaluation and suggestions. We have addressed the specific comments as below.

Reviewer #1: Minor issues
Line 31: In the abstract, “infection-driven” chronic kidney disease is mentioned, but this is the only time “infection-driven” is mentioned in the manuscript. Would you please clarify whether this paper is about infection-driven CKD or CKD of any aetiology. If genuinely “infection-driven” CKD, please clarify in the author summary and the manuscript eg in line 116.

Response: We apologize for this lack of clarity. We have revised as follows:

In abstract: “*Strongyloides stercoralis* infection typically causes severe symptoms in immunocompromised patients. This infection can also alter the gut microbiota and is often found in areas where chronic kidney disease (CKD) is common. However, the relationship between S. stercoralis and the gut microbiome in chronic kidney disease (CKD) is not understood fully. Recent studies have shown that gut dysbiosis plays an important role in the progression of CKD. Hence, this study aims to investigate the association of S. stercoralis infection and gut microbiome in CKD patients.”

The author summary and introduction have been revised in line with this.

Line 125: renal infection with other intestinal parasites. Should this be “renal infection, infection with other parasites.”?

Response: We intended to say that subjects were excluded if they had intestinal parasites other than *S. stercoralis*. Participants in the Ss- group were free of all intestinal parasites and the Ss+ group were infected only with *S. stercoralis*.

The text as been revised as follows: “Exclusion criteria were as follows: use of antibiotics or probiotics, diabetes, autoimmune disease, urinary tract infection and infection with intestinal parasites other than S. stercoralis.”

In Table 1, eosinophilia is elevated in both the Ss+ and Ss- groups, which suggests that there were other helminth infections in the Ss- group. Would you please explain this anomaly?

Response: Elevation of eosinophils may not only cause by parasitic infection but also other allergen-induced allergy. This may increase eosinophils in Ss- group.

Line 208: Is the number of species correct here? The number should be more than or equal to the number of genera.

Response: The number of species was less than the number of genera because some taxa were only identified to genus. In this study, we did not do whole-genome sequencing, thus, not all bacterial species could be identified. We only sequenced the V3-V4 region of 16s rRNA.

Line 211-212: State in the text which group had the greater beta diversity [the Ss- group, according to Fig 2].

Response: We stated that “The unweighted UniFrac distances, reflecting beta diversity, were significantly greater in the Ss− group than the Ss+ group (*P* = 0.00019).” This means that the beta diversity was greater in the Ss- group.

Minor grammatical, spelling or punctuation changes:
Line 72: change “significant” to “significantly”
Line 116: change “age) of” to “age) with”
Line 220: change “significantly” to “significance”
Line 306: change “Anearostipes” to “Anaerostipes”
Line 583 (Caption for Fig 3): change “Oder” to “Order”
Line 618 (Caption for Fig 7): insert “;” after “age”

Response: We have corrected accordingly in the revised version.

Reviewer #1: To my knowledge, this study is the first to show in CKD patients that S. stercoralis infection is associated with decreased diversity of the faecal microbiota, increased abundance of pathogenic microbial genera and decreased abundance of SCFA-producing genera. These are very important findings.
Although the study involved a small number of participants, the use of matched pairs ensured that the S. stercoralis-infected group was similar to the uninfected group in characteristics other than S. stercoralis infection status. PCR testing was used to ensure as much as possible that the uninfected group was truly S. stercoralis-free. The authors were careful to ensure that there was sufficient DNA in the extracted samples to carry out gene sequencing.

Although the manuscript states that the study protocol was approved by the human ethical review committee of Khon Kaen University (HE631200), the manuscript does not state whether informed consent was obtained from the participants. Details about informed consent should be included in the manuscript.
Response: We have added the details about informed consent as follows: “Informed consent was obtained from all participants under the CKD project [14].”

Reference

14. Cha'on U, Wongtrangan K, Thinkhamrop B, Tatiyanupanwong S, Limwattananon C, Pongskul C, et al. CKDNET, a quality improvement project for prevention and reduction of chronic kidney disease in the Northeast Thailand. BMC Public Health. 2020;20(1):1299. Epub 2020/08/29. doi: 10.1186/s12889-020-09387-w. PubMed PMID: 32854662; PubMed Central PMCID: PMCPMC7450931.
